# A comparative study of standardized quantitative and visual assessment for predicting tumor volume and outcome in newly diagnosed diffuse large B-cell lymphoma staged with 18F-FDG PET/CT

**DOI:** 10.1186/s13550-019-0503-z

**Published:** 2019-05-03

**Authors:** Lars C. Gormsen, Mikkel H. Vendelbo, Mette Abildgaard Pedersen, Ate Haraldsen, Karin Hjorthaug, Trond Velde Bogsrud, Lars J. Petersen, Karen Juul Jensen, Rasmus Brøndum, Tarec C. El-Galaly

**Affiliations:** 1 Department of Nuclear Medicine & PET Centre, Aarhus University Hospital, DK 8000 Aarhus C, Aarhus, Denmark; 2 Department of Hematology, Aalborg University Hospital, Aalborg, Denmark; 3 Department of Nuclear Medicine, Clinical Cancer Research Center, Aalborg University Hospital, Aalborg, Denmark; 4 Department of Hematology, Odense University Hospital, Odense, Denmark; 5 Department of Clinical Medicine, Aalborg University, Aalborg, Denmark; 6 PET Centre, University Hospital of North Norway, Tromso, Norway; 7 Department of Biomedicine, Aarhus University, Aarhus, Denmark

**Keywords:** DLBCL, MTV, 18F-FDG PET/CT, Prognosis

## Abstract

**Background:**

Semi-automated quantitative measurement of metabolic tumor volume (MTV) for prognosis in diffuse large B-Cell lymphoma (DLBCL) has gained considerable interest lately. However, simple tumor volume measures may be inadequate for assessment of prognosis in DLBCL as other characteristics such as growth pattern and metabolic heterogeneity may be just as important. In addition, MTV measurements require delineation of tumor lesions by semi-automated software, which can be time-consuming. We hypothesized that a simple visual assessment of tumor volume performs as well as standardized MTV measurements in DLBCL prognostication.

**Materials and methods:**

Quantitative and visual analyses of pre-therapy 18F-FDG PET/CT scans in 118 patients with newly diagnosed DLBCL were conducted. Quantitative analyses were performed using Hermes TumourFinder® to obtain MTV^2.5^ (SUV 2.5 cut-off) and MTV^41^ (41% SUVmax isocontour cut-off). Visual assessments included a binary prediction (good/poor prognosis) as well as tumor burden based on a visual analog scale (MTV^VAS^) and an estimated volume (eMTV). Three experienced nuclear medicine physicians who were blinded to clinical outcome performed visual evaluations. Progression-free survival was evaluated by Kaplan-Meier curves and log-rank test. Inter-observer variability was evaluated by Fleiss’ kappa for multiple observers.

**Results:**

In the quantitative analysis, a ROC-determined MTV^2.5^ cut-off (log-rank *p* = 0.11) seemed to outperform MTV^41^ (log-rank *p* = 0.76) for PFS prediction. TLG2.5 (log-rank *p* = 0.14) and TLG41 (log-rank *p* = 0.34) were not associated with outcomes. By visual analysis, all three reviewers were able to stratify patients into good/poor prognosis (reviewer A log-rank *p* = 0.002, reviewer B log-rank *p* = 0.016, and reviewer C log-rank *p* = 0.012) with fair inter-observer agreement (Fleiss’ kappa 0.47). MTV^VAS^ and eMTV were not consistently correlated with the outcome.

**Conclusion:**

Predictions of outcome after first-line treatment for DLBCL were surprisingly good when left to the unsupervised, subjective judgment of experienced readers of lymphoma 18F-FDG-PET/CT. The study highlights the importance of non-standardized clinical judgments and shows potential loss of valuable prognostic information when relying solely on semi-automated MTV measurements.

## Background

Diffuse large B-cell lymphoma (DLBCL) is the most common type of non-Hodgkin lymphoma in the Western world. The majority of patients with DLBCL respond to standard immunochemotherapy with a combination of the drugs rituximab, cyclophosphamide, doxorubicin hydrochloride (hydroxydaunorubicin), vincristine sulfate, and prednisone (R-CHOP), but 30–40% of the patients are refractory or relapse following initial response [1]. These patients have dismal outcomes and only a minority can be cured by salvage high-dose therapy and autologous stem cell transplantation [2, 3]. No major progress has been made in the treatment of DLBCL since the introduction of rituximab. Attempts to improve outcomes by intensifying chemotherapy and rituximab-dosing schedules have failed [4–10]. A treatment escalation strategy based on early PET/CT response did not improve outcomes in poor responders over and above what was achieved by continuing standard therapy [11]. Also, the predictive value of interim PET/CT in DLBCL, unlike in Hodgkin lymphoma [12], remains controversial [11]. The key to improving outcomes in DLBCL may reside in better risk stratification of DLBCL using an integrative approach that combines baseline clinical risk factors, genomic features, and early treatment response [13, 14]. Combinations of tumor volume measurements on baseline PET/CT and early treatment response have been proposed to identify high-risk DLBCL patients likely to fail standard therapy [14]. Providing metabolic tumor volume measurements (quantitative PET/CT), however, is time-consuming and requires highly standardized algorithms for image acquisition and reconstruction. Furthermore, relying increasingly on “automatization” for prognostics minimizes the influence of clinical judgments by readers with years of experience. Numerous automated or machine learning-based algorithms for outcome prediction, particularly in radiology have been proposed in the past decade; however, only few have been found to be on par or outperform human-level performance [15]. To test the superiority of quantitative PET/CT over simple and unsupervised assessment of prognosis by experienced nuclear medicine specialists, we compared the prognostic value of both methods in a consecutive cohort of 118 patients.

## Methods

One hundred and eighteen patients with newly diagnosed DLBCL treated with R-CHOP/R-CHOP-like therapy and undergoing diagnostic work-up including baseline 18F-FDG PET/CT in the period 2007–2012 at Aalborg University Hospital were included in this retrospective study. Clinical information including outcome parameters was collected from medical records. The Danish Data Protection agency (ref 1-16-02-88-15) and the Danish Ministry of Health approved the study (ref 3-3013-860/1/).

### PET/CT acquisition

Staging 18F-FDG PET/CT scans were carried out after a 6-h fast on a GE Discovery VCT 710 integrated PET/CT system in accordance with local protocols and manufacturer guidelines. Images from the base of the skull to the proximal thigh were acquired a median of 71 (range 57–166) minutes after injection of 4 MBq/kg 18F-FDG and were reconstructed using iterative reconstruction.

### Quantitative image analysis (Table 1)

18F-FDG PET/CT images were analyzed by author LCG using Hybrid Viewer (Hermes Medical Solutions, Sweden) with the plug-in TumourFinder. Lymphoma lesions were segmented using both a SUV > 2.5 threshold (SUV^2.5^) as well as > 41% of the SUVmax (SUV^41^) thresholding. Quantitation parameters metabolic tumor volume (MTV^2.5^ and MTV^41^) and total lesion glycolysis (TLG^2.5^ and TLG^41^) were recorded for both segmentation methods.

**Table 1:** Semi-automated methods and visual assessment of tumor volume and prognosis

Method	Semi-automated measurements		Visual assessment		
	MTV^2.5^	MTV^41^	eMTV	MTV^VAS^	Prognosis
Description	Metabolic tumor volume delineation > SUV 2.5	Metabolic tumor volume delineation > 41% of SUVmax	Metabolic tumor volume assessed visually	Degree of metabolic tumor volume assessed on VAS scale	Prognosis assessed visually based on volume, heterogeneity, involvement of extra-nodal organs
Parameter	Continous (ml)	Continous (ml)	Continous (ml)	Continous (1–9)	Dichotomous (poor/favorable)

### Visual image analysis (Table 1)

**Fig. 1:**
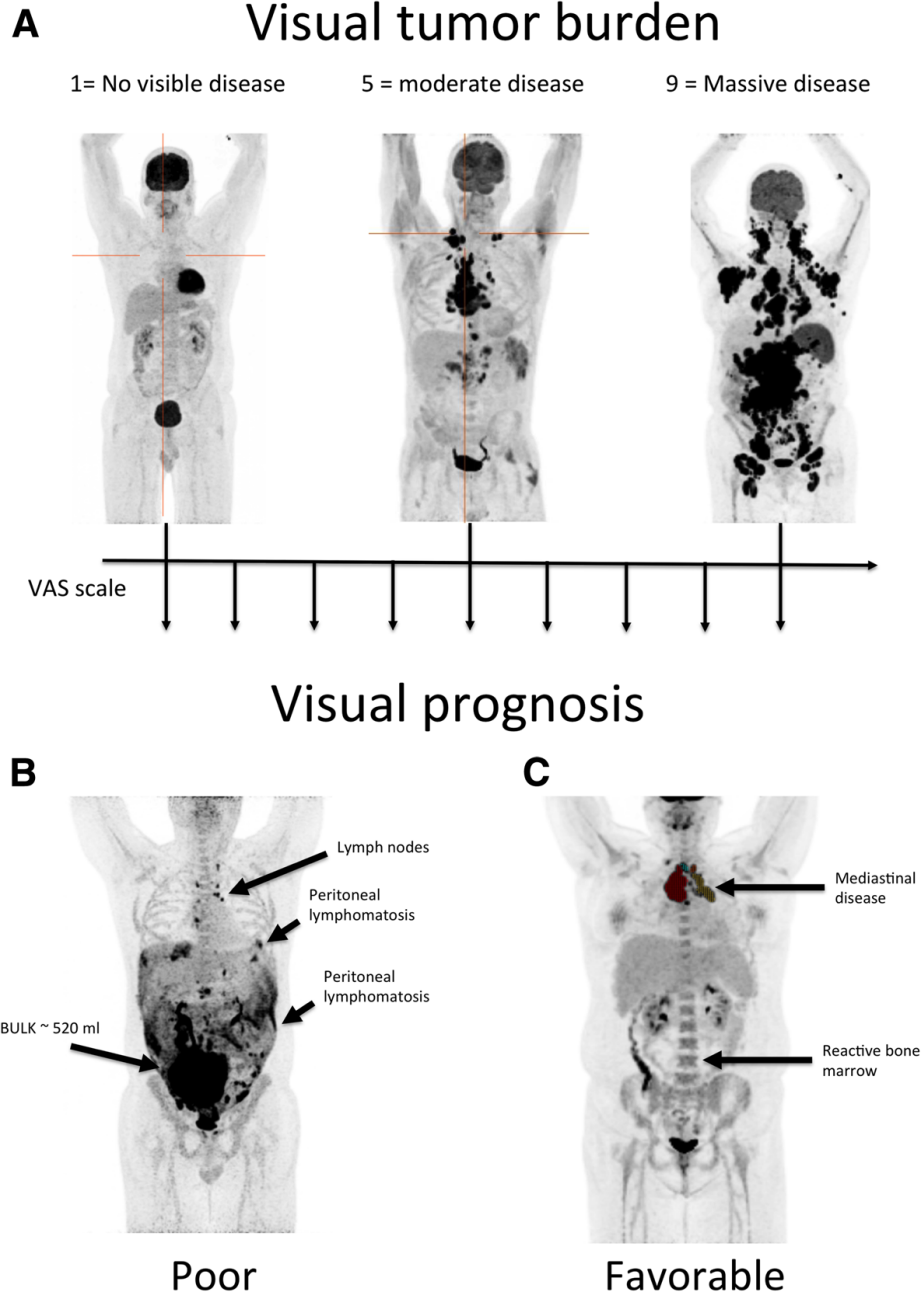
Metabolic tumor burden assessed on a VAS scale with representative examples of patients graded as 1, 5, and 9 (**a**). All reviewers used these examples to grade individual cases in the study. **b** An example of a patient with a poor prognosis based on the widespread peritoneal lymphomatosis, bulky disease, and high metabolic tumor volume. Pointedly, the peritoneal lymphomatosis may be difficult to accurately quantify using semi-automatic tumor delineation tools whereas the severity of disease is readily perceived by visual inspection using a maximum intensity projection FDG PET/CT. **c** A patient with limited mediastinal disease used as an example of a patient with favorable prognosis

Three experienced (> 10 years) nuclear medicine physicians (A.H., T.V.B., and K.H.) blinded to clinical information except patient age performed visual analysis of images. Observers were asked to rate the following tentatively predictive parameters: (1) prognosis (poor/favorable), (2) tumor volume estimate (eMTV) (in milliliters), and (3) tumor volume (MTV^vas^) (visual analog score; VAS, 1–9). Observers were asked to restrict the time spent evaluating these parameters to a maximum of 30 s per evaluation in order to reflect the intuitive interpretation of the images. Figure 1 depicts the VAS scale used by our reviewers as well as examples of patients with poor and favorable prognosis. To visually assess prognosis, reviewers were asked to take into account tumor heterogeneity, tumor volume, infiltration into adjoining tissue, extra-nodal spread, and diffuse involvement of, e.g., the pleura or peritoneum.

In case of disagreement between reviewers, prognosis was analyzed as a trichotomous variable (favorable/intermediate/poor), whereas eMTV and MTV^vas^ were averaged for all reviewers.

## Statistics

Fleiss’ kappa was used to evaluate the inter-observer variability when reporting the subjective assessment of prognosis, while the variability for MTV^vas^ and eMTV was evaluated using the intra-class correlation (ICC). For MTV^VAS^, we used the ICC described in [16] while the ICC for absolute agreement between a random sample of readers was used for eMTV (ICC2 from the R-package *psych*). The Pearson correlation coefficient was used to measure consistency between MTV values obtained by the automated software (MTV^2.5^ and MTV^41^) and visual estimates (eMTV).

Survival analyses were performed according to prognosis subjectively assessed (good/bad), eMTV, MTV^vas^, MTV^2.5^, MTV^41^, TLG^2.5^, and TLG^41^. Progression-free survival (PFS) was defined as the time from diagnosis to relapse/progression or death from any cause. Overall survival (OS) was defined as the time from diagnosis to death from any cause. Optimal PFS cut-offs for continuous variables were determined with receiver operating characteristics (ROC) curves for three-year survival and three-year progression-free survival as the point with the shortest distance from (0, 1). Survival curves were computed using the Kaplan-Meier method and differences were tested using the log-rank test. Statistics were calculated using R version (3.4.3).

## Results

**Fig. 2:**
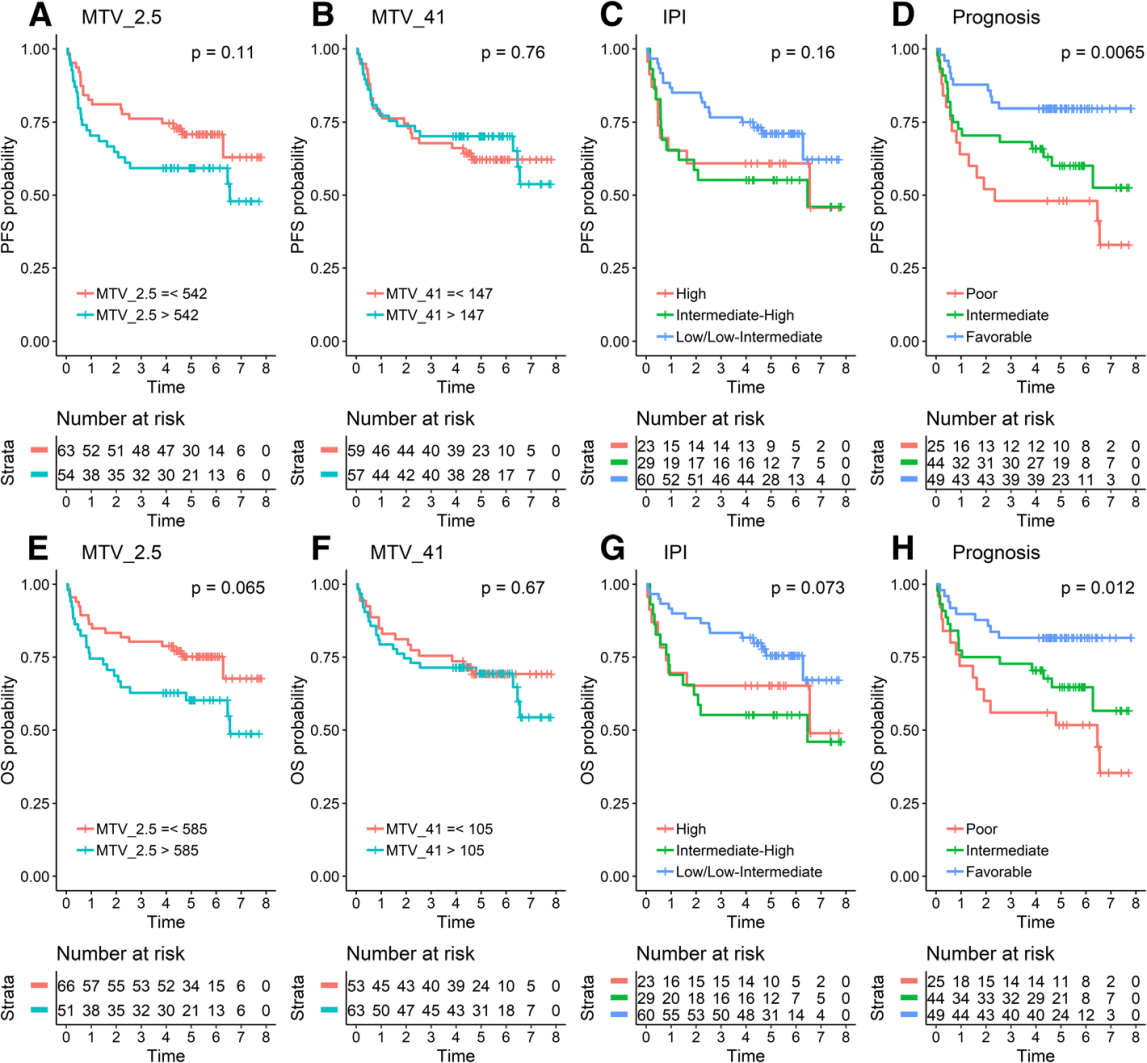
Progression-free survival (PFS) and overall survival (OS) using automated metabolic tumor volume (MTV) measurements (**a**, **b**, **e, f**), IPI (**c, g**) vs. visual assessment of prognosis by expert readers (**d, h**). As seen, the visual PET assessment of prognosis significantly outperformed both the automated PET metrics as well as the prognosis by the treating hematologist (IPI)

A total of 118 patients (76 males and 42 females) with median age 66 (range 18–88) were included (Table 2). According to the international prognostic index (IPI), 60 patients (50.8%) were low/low-intermediate risk, 29 (24.6%) were high-intermediate, and 23 (19.5%) were high-risk. Information on IPI was not available in 5 patients. Median follow-up was 67 months (reverse Kaplan-Meier method) and the overall 5-year PFS and overall survival (OS) estimates were 65.7% and 68.9%, respectively (Fig. 2 for IPI-specific survival curves). Treatment regimens were R-CHOP (*n* = 104, 88.1%), R-CHOEP (*n* = 12, 10.2%), and R-CEOP (*n* = 2, 1.7%). Two patients turned out to be CD20 negative and thus had rituximab removed from their treatment regimens.

**Table 2:** IPI classification for patients. Pre-therapy IPI was unavailable in five patients

Patient characteristics	
Age	66 (16–88)
Ann Arbor I–II	40 (34%)
Ann Arbor III–IV	76 (64%)
Nodal > 1	31 (26%)
ECOG > 1	18 (15%)
LDL > UNL	62 (53%)
IPI > 2	52 (44%)

The inter-observer agreement evaluated by Fleiss’ kappa was moderate at 0.47 when PET/CT readers assessed prognosis (poor/favorable), whereas the agreement for MTV^vas^ and eMTV were good to excellent with values of 0.81 and 0.66, respectively. Finally, moderate to high correlations between automated MTV measurements (MTV^2.5^, MTV^41^, TLG^2.5^, and TLG41) and visual eMTV estimates were found, as shown in Table 1. There was, however, no unanimous highest correlation between the automated and visually assessed MTV across the three reviewers.

### Prediction of outcome using automated software

The areas under the ROC curves for 3-year PFS predictions were 0.59, 0.54, 0.58, and 0.56 for MTV^2.5^, MTV^41^, TLG^2.5^, and TLG^41^, respectively. The corresponding values for 3-year OS predictions were 0.58, 0.53, 0.57, and 0.54 for MTV^2.5^, MTV^41^, TLG^2.5^, and TLG^41^, respectively. The best dichotomous cut-off values for PFS based on tumor burden were 542 ml, 147 ml, 3237, and 1485 for MTV^2.5^, MTV^41^, TLG^2.5^, and TLG^41^, respectively. Corresponding values for OS were 585 ml, 105 ml, 3560 ml, and 2557 ml for MTV^2.5^, MTV^41^, TLG^2.5^, and TLG^41^, respectively. Using these thresholds, only MTV^2.5^ was borderline associated with OS (log-rank *p* = 0.065) and PFS (log-rank *p* = 0.11) (Fig. 2a and e), whereas MTV^41^ was not associated with OS (log-rank *p* = 0.76) or PFS (log-rank *p* = 0.67) (Fig. 2b and f). TLG2.5 (log-rank *p* = 0.14) or TLG41 (log-rank *p* = 0.34) was not prognostic in PFS or OS analyses.

### Visual estimation of tumor volume

For MTV^vas^ and eMTV, the areas under ROC curves for the three reviewers for PFS and OS were 0.62, 0.63, and 0.58 and 0.61, 0.61, and 0.56 for MTV^vas^ and 0.60, 0.62, and 0.58 and 0.60, 0.60, and 0.56 for eMTV. Best cut-offs for MTV^vas^ were 4, 4, and 6 for both PFS and OS. Best eMTV cut-off for high and low tumor burden was 288, 450, and 425 ml for PFS and 288, 450, and 1250 ml for OS. Subsequent Kaplan-Meier analyses showed that only the eMTV threshold for one of the reviewers (450 ml) could separate patients in high or low risk (Kaplan-Meier log-rank *p* = 0.034 for PFS). However, the areas under the ROC curves were comparable or better for eMTV than for MTV. Table 3 shows the correlation between eMTV of individual reviewers and the semi-automated measurements, which was surprisingly good for two reviewers.

**Table 3:** Pearson correlation between visually assessed eMTV and semi-automated measurements

eMTV—Pearson correlation							
	Reviewer 1	Reviewer 2	Reviewer 3	MTV^2.5^	MTV^41^	TLG^2.5^	TLG^41^
Reviewer 1	1.00	0.66	0.66	0.90	0.82	0.82	0.72
Reviewer 2	0.66	1.00	0.69	0.80	0.74	0.89	0.84
Reviewer 3	0.66	0.69	1.00	0.68	0.68	0.77	0.76
MTV^2.5^	0.90	0.80	0.68	1.00	0.92	0.89	0.79
MTV^41^	0.82	0.74	0.68	0.92	1.00	0.80	0.81
TLG^2.5^	0.82	0.89	0.77	0.89	0.80	1.00	0.95
TLG^41^	0.72	0.84	0.76	0.79	0.81	0.95	1.00

### Visual prognosis

The subjective visual assessments of prognosis (poor/favorable) were highly associated with outcomes both in PFS and OS analyses for all three reviewers, and the consensus prognosis for the three reviewers (all poor/mixed/all favorable) also showed strong association (*p* < 0.01 for PFS and *p* = 0.01 for OS (Fig. 2d and g). For the 44 patients in the mixed group, the majority vote was “poor” for 14 and “favorable” for the remaining 30. Ranges of MTV2.5 were 287–8462, 8–2672, and 14–2260 for “all poor,” “all favorable,” and “mixed” consensus prognosis patients, respectively. The overlap in tumor volume between patients classified by visual prognosis underlines the importance of other factors than tumor burden, when reviewers assessed prognosis.

**Fig. 3:**
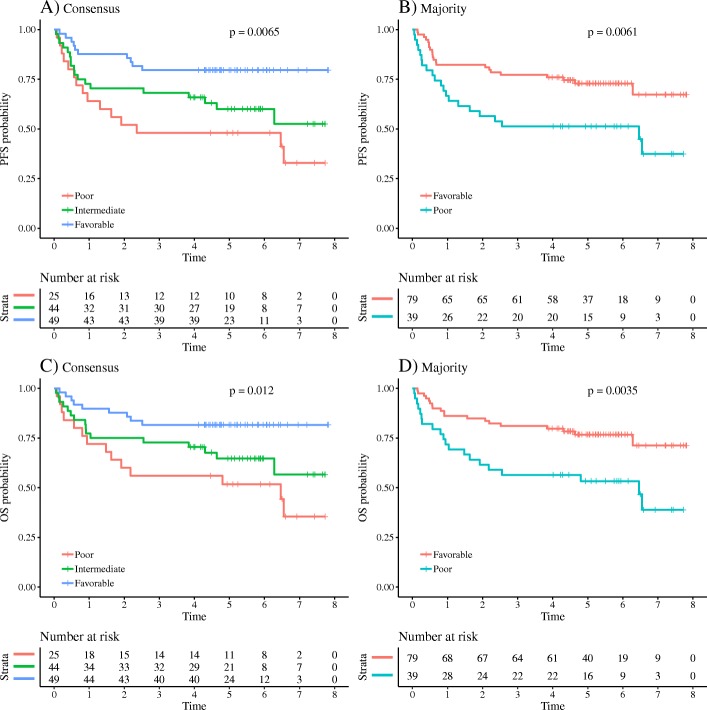
Progression-free survival (PFS) and overall survival (OS) using the visual assessment of prognosis by expert readers. **a**, **c** The consensus diagnosis of the three readers with an intermediate group reflecting disagreement between reviewers. **b**, **d** Prognosis based on majority decision. As seen, both consensus and majority decisions were able to significantly identify high- and low-risk patients

Using the majority vote, the log-rank test also showed significant differences between the two groups, differences in 2-year survival were, however, larger when including a mixed group, with differences between favorable and poor of respectively 28% and 36% for OS and PFS using the consensus diagnosis and differences of 23% and 26% for OS and PFS using the majority vote (Fig. 3).

## Discussion

Understanding of DLBCL and the outcome diversity has evolved substantially over the last decade, in particular with the recognition of distinct and prognostic molecular genetic subtypes of DLBCL [13, 17], although none of these classifications have well-established influence on treatment decisions [18–21]. In parallel, the use of staging 18F-FDG PET/CT has enabled a more precise estimate of total tumor volume, one of the historically most important prognostic factors in lymphoma usually determined indirectly by the Ann Arbor stage and/or LDH level. Not surprisingly, most [14, 22–24] but not all [25] studies have demonstrated tumor burden as an adverse prognostic factor regardless of how the lymphoma lesions are delineated. This has led to increased interest in using automated measurements of pre-therapy MTV to guide treatment decisions, occasionally in combination with already established response assessment criteria [14].

There are several examples of successful automated scoring systems to report disease severity in the field of pathology and radiology, e.g., Ki-67 index [26], brain atrophy by MRI [27], breast density by mammography [28]. In general nuclear medicine, automatic scoring of stress-induced myocardial ischemia by myocardial perfusion imaging has been shown to perform as well as visual interpretation [29, 30]. However, in order to replace expert analysis in routine practice, an automated technique should either outperform expert analysis or perform as well but with less human effort to avoid reader fatigue. Currently, full measurement of lymphoma MTV has some drawbacks that limit clinical implementation: First, MTV measurements are time-consuming using available software solutions. Second, consensus regarding the best method for lymphoma lesion delineation has not been reached. Third, it remains to be shown that MTV measurements in DLBCL add prognostic information over and above what can be determined from simple visual image assessment and commonly used clinical risk scores.

We used Hermes TumorFinder to evaluate the two most commonly used lesion delineation algorithms: one based on a fixed threshold of SUV2.5 (MTV^2.5^) [14] and one using a 41% of SUVmax isocontour (MTV^41^) [31]. Both methods require manual adjustments of segmentations provided by the software (e.g., cropping out of the kidneys, ureters, bladder, liver, heart, and brain), which took 10–15 min on average per patient, similar to what was reported in a previous study [32]. As detailed in Fig. 2, MTV^2.5^ performed significantly better than MTV^41^ in predicting PFS and OS, although we were unable to separate high- and low-risk groups as well as previously reported [14, 22–24]. This discrepancy may well be explained by the ROC-based MTV^2.5^ cut-off for high tumor burden, which in our cohort was approximately 550 ml. Others have reported markedly lower cut-off values of ~ 400 ml [32] and 220 ml [24]. Importantly, our cohort was relatively small and weak prognostic factors may have been missed due to the risk of type II errors.

Our expert readers outperformed the automated prediction of outcome when using all available information from the PET/CT scans including visually estimated MTV, heterogenous lesion pattern, and extra-nodal involvement in a binary score (good/poor prognosis). Moreover, they did so in less than a minute per patient and therefore with far less effort and without the need for additional software packages than a standard PET/CT viewer. Interestingly, the three-decade old IPI classification based on simple clinical risk factors was as prognostic as the automated MTV, although not on par with the binary assessment of prognosis provided by the expert readers (Fig. 2).

The present study also points out the flaws of visual image interpretation, in particular inter-observer variability. When asked to grade metabolic tumor volume on a VAS, agreement among reviewers was poor (Fleiss’ kappa 0.28) and the consensus MTV^vas^ could not satisfyingly stratify patients into high- or low-risk groups. Our reviewers were also unable to estimate absolute MTV in milliliters (eMTV) to a level that reflected true measured levels. In addition, although individual reviewers were able to significantly prognosticate outcome on a dichotomous scale (poor/favorable prognosis), agreement among reviewers was only moderate (Fleiss kappa 0.47). In other words, our reviewers did not uniformly identify the same patients as having a good or poor prognosis.

## Conclusions

In summary, automated MTV measurements in DLBCL are time-consuming and may not add prognostic information over and above what is delivered in an unsupervised expert analysis, which on the other hand is prone to inter-reader variability and limited generalizability. Importantly, this study is not intended to challenge, devaluate, or oppose the important progress made in the use of quantitative PET/CT in lymphoma. However, it highlights the fact that any prognostic information based on automated measures should be held up against the information obtained in an unsupervised clinical judgment based on physician experience. In fact, a general reminder that algorithms do not always outperform good clinical judgment and that their value should be tested against the unmeasurable knowledge of trained clinical experts.

## Abbreviations

DLBCL: Diffuse large B-cell lymphoma
ECOG: Eastern Cooperative Oncology Group
eMTV: Visually estimated MTV (ml)
ICC: Intra-class correlation
IPI: International prognostic index
MRI: Magnetic resonance imaging
MTV: Metabolic tumor volume
MTV^2.5^: Semi-automated MTV based on SUV 2.5 threshold
MTV^41^: Semi-automated MTV based on 41% of SUV max threshold
MTV^vas^: Visually assessed tumor volume (0–9)
OS: Overall Survival
PFS: Progression-free survival
R-CHOP: Rituximab, cyclophosphamide, doxorubicin hydrochloride (hydroxydaunorubicin), vincristine sulfate, and prednisone
ROC: Receiver operating characteristics
TLG: Tumor lesion glycolysis
TLG^2.5^: Semi-automated TLG based on SUV 2.5 threshold
TLG^41^: Semi-automated TLG based on 41% of SUV max threshold
